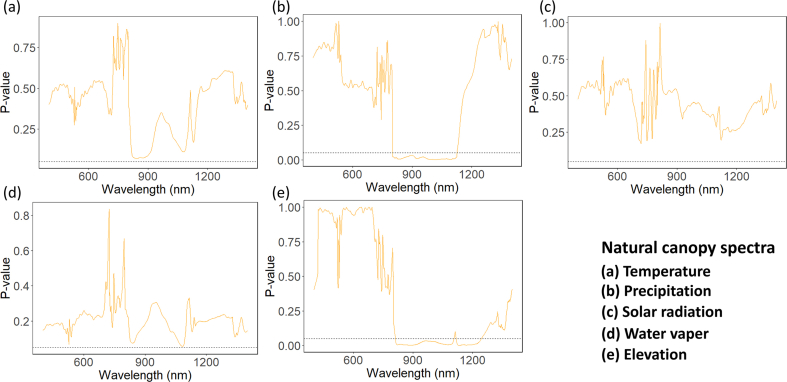# Genetic constraints on in-situ reflectance spectral variation in bermudagrass populations across Hainan Island

**DOI:** 10.1016/j.plaphe.2026.100168

**Published:** 2026-01-14

**Authors:** Jingxue Zhang, Jiali Shang, Jiangui Liu, Liwen Wang, Mengli Han, Minghui Chen, Chen Chen, Yuhong He, Xuebing Yan

**Affiliations:** a College of Animal Science and Technology, Yangzhou University, Yangzhou, 225000, China; b Department of Geography, Geomatics and Environment, University of Toronto Mississauga, Mississauga, L5L 1C6, Canada; c Ottawa Research and Development Centre, Agriculture and Agri-Food Canada, Ottawa, K1A0C6, Canada

**Keywords:** Leaf-level spectra, Genetic differences, Hyperspectral remote sensing, Biodiversity, *Cynodon dactylon*

## Abstract

Remote sensing is increasingly applied in monitoring biodiversity and ecosystem health, however, its utility for detecting heritable aspects of biodiversity is limited by the challenge of distinguishing genetically adapted traits from plastic phenotypic responses. The gap between genetic information and spectral traits is from the indirect, environment-mediated nature of reflectance signals. In this study, we acquired hyperspectral reflectance data (350–2500 nm) combined with specific-locus amplified fragment sequencing (SLAF-seq) across wild bermudagrass (*Cynodon dactylon*) populations on Hainan Island and experimental common garden to investigate genetic constraints on spectral variation. We assessed relationships between genetic information and spectral signatures through Partial Mantel Tests and Partial Least Squares Discriminant Analysis (PLS-DA). Leaf spectral-genetic correlations (r = 0.4–0.7; P < 0.05) were detected significantly in the SWIR region between 1900 and 2400 nm, underscoring the role of ecological heterogeneity in shaping adaptive spectral traits. Environmental covariates including annual mean temperature, total annual precipitation, elevation, mean solar radiation, mean water vapor content, presented significant liner correlation (r > 0.4, p < 0.05) with leaf spectral-genetic similarity, which revealed that we disentangled SWIR bands as heritable spectral signals from plastic phenotypic responses. PLS-DA achieved a higher accuracy in classifying populations using natural leaf spectra (Accuracy = 86.76 %; Kappa = 0.86) than using canopy data (Accuracy = 41.74 %; Kappa = 0.37), which is influenced by canopy structure. Our findings highlight that integrating spectral-genetic similarity with environmental drivers can identify inheritable spectral signals, providing a pathway for monitoring the adaptive evolution of biodiversity using remote sensing under rapid global change.

## 1 Introduction

Remote sensing has emerged as a powerful tool for large-scale biodiversity monitoring, offering significant potential for assessing ecosystems under environmental change by overcoming the spatial and temporal limitations of ground-based surveys [[1], [2], [3]]. Both hyperspectral and multispectral remote sensing contribute to biodiversity monitoring by capturing spectral information that reflects plant physiological, biochemical traits, canopy structure, and species assemblages. Multispectral sensors track species distributions, vegetation cover change, and ecosystem processes across landscapes by offering coarser spectral resolution but broader spatial or temporal coverage [4]. While hyperspectral remote sensing capture fine-scale biodiversity by detecting subtle, spectrally distinct signatures linked to physiological, biochemical, or morphological adaptations, supporting assessments of functional and sometimes even genetic diversity [5]. Here, we use the term biodiversity primarily in reference to adaptive genetic and functional diversity across plant populations, as these dimensions underpin ecosystem resilience to global climate change and can be indirectly monitored through spectral signatures associated with growth dynamics and resource-use strategies [6,7].

Remote sensing quantifies species adaptability through heritable spectral traits that serve as indicators of adaptive potential under environmental changes [8]. Spectral signatures capture phenotypic traits that represent the ecological expression of underlying genetic diversity and thus provide a pathway for linking genetic information with remotely sensed data [9]. Emerging machine learning and deep learning approaches are transforming the field of plant phenotyping. For example, advanced convolutional neural networks (CNNs) and other deep learning architectures have shown significant success in complex phenotyping tasks by effectively learning hierarchical spectral-spatial features from plant data [10,11]. Furthermore, user-friendly tools such as CountShoots software have been developed, which utilizes models like YOLOX and the Slash Pine Shoot Counting Network (SPSC-net) to efficiently estimate new shoot density from unmanned aerial vehicle imagery [12]. Collectively, these advances underscore a methodological shift toward highly predictive, automated models in plant phenomics. Despite these advances, genetic diversity, an Essential Biodiversity Variable (EBV) critical for species adaptability in response to environmental changes, remains understudied in remote sensing community [13]. Traditional genetic methods, such as DNA sequencing or microsatellite analysis, are effective but impractical for large-scale studies spanning multiple sites due to cost and time constraints [14]. To bridge this gap, integrating remote sensing with genetic data has emerged as a promising approach to mapping biodiversity [15].

Reflectance spectroscopy is particularly valuable for linking genetic and phenotypic variation. Vegetation spectra are fundamentally determined by leaf pigments, structural characteristics, and water content, with genetic architecture shaping these traits and environmental conditions modulating their expression [16,17]. This makes spectroscopy as a powerful tool for assessing plant phylogenetic and functional diversity across spatial and temporal scales [18,19]. The ability of spectroscopy to capture subtle spectral signatures has attracted the attention from plant geneticists, who are exploring its potential for assessing genetic variation remotely. For example, genetic differences in chlorophyll content or leaf structure can manifest as distinct spectral signatures, enabling remote detection of intraspecific variation [20].

Hyperspectral remote sensing allows for accurate classification (94 %) of globally dominant grass lineages (C3 Pooideae and C4 Andropogoneae/Chloridoideae), supporting its potential use in phylogenetically informed biodiversity mapping [21]. While studies have successfully linked spectral data to genetic differentiation in taxa like *Populus tremuloides* [22] and *Populus fremontii* [23] across wild populations, challenges persist in that, spectral-genetic correlations are often subtle within species, context-dependent, and influenced by environmental heterogeneity [24,25]. Environmental variability influences the correlation between spectral signatures and genetic variation across spatial scales. Local environmental conditions can alter trait expression, making it complex to disentangle genetic adaptation from phenotypic plasticity. Recent studies indicated the potential of spectral reflectance as a proxy for genetic diversity, by revealing patterns of adaptation to local environmental conditions [26,27].

This study integrates multi-scale spectral data (leaf and canopy), population genomic information, and environmental data to investigate the links between spectral trait variation and genetic diversity in wild bermudagrass populations on Hainan Island. Prior researches have predominantly utilized spectral data to discern spectral and genetic differences between species for community-level applications, its application to resolve intraspecific genetic structure remains largely unexplored. We address this gap by establishing a direct, population-level link between spectral variation and genetic divergence, offering new insights into micro-evolutionary processes such as local adaptation. Hainan Island, an area of high environmental heterogeneity, is selected as our study system, which provides a natural experimental setting to test how environmental gradients drive intraspecific spectral trait differentiation—a context less easily achieved in continental landscapes. In addition, to disentangle genetic from environmental effects, we collected three complementary spectral datasets: from leaves in natural habitats, from corresponding canopies, and from a common garden where environmental noise is minimized. This design allows us both to quantify heritable spectral signals and to assess their scalability to canopy-level sensing. This design allows us both to quantify heritable spectral signals and to assess their scalability to canopy-level sensing. Building on prior knowledge of population genetic structure in this system [28], we specifically ask: Do different environmental conditions have a direct plastic effect or a selective genotype effect on spectral variation among populations on Hainan Island? To address this question, using leaf and canopy spectroscopy, we acquired spectral and genomic datasets across wild populations and experimental common garden to investigate genetic constraints on spectral variation in bermudagrass across Hainan Island. The objectives of this study were to: (1) investigate geographic patterns of spectral variation among populations; (2) assess spectral-genetic relationship and environmental effects; (3) evaluate the ability of spectral reflectance in distinguishing populations and genotypes. We aim to advance spectroscopic techniques as scalable tools for monitoring genetic diversity, informing conservation strategies, and understanding adaptive responses to environmental change.

## 2 Materials and methods

### 2.1 Study area

Hainan Island, located in the southernmost part of China, is renowned for its tropical climate, diverse ecosystems, and rich biodiversity [29]. The island features a range of environmental conditions, including differences in soil type, moisture levels, elevation, and sun exposure, which offers it an ideal setting to explore the variability in these aspects. Bermudagrass (*Cynodon dactylon*) exhibits significant phenotypic and genetic variation across different environments. It is widely used for home lawns, athletic fields, golf course turf, parks, recreational areas, and roadside vegetation [30]. The combination of Hainan Island's diverse environmental gradients and Bermudagrass's extensive ecological and genetic variability provides a unique opportunity to assess how environmental factors shape spectral-genetic similarity.

Samples were collected under permit issued by Hainan Provincial Authorities. We collected spectral and genomic datasets from 14 geographically diverse sites for comprehensively analyzing the spectral and genetic variation of bermudagrass among 14 populations across Hainan Island, China (Fig. S1). The island covers an area of approximately 33,920 square kilometers, and is surrounded by the South China Sea. The 14 geographic sites include Mingshan, Tianwei, Houhu, Lanyang, Wene, Haiwei, Nalaimiao, Kanmao, Nanfeng, Chongmentou, Lianshan, Yongming, Yinggehai, and Linxin (Table S1). These sites represent a range of diverse environmental conditions, with a wide variance in both spectral and genetic diversity in plants. The sites span from coastal lowlands (e.g., Linxin, Tianwei) to mountainous interiors (Nalaimiao, Kanmao, Nanfeng). Elevation ranges from sea level (Mingshan) to around 1800 m (Chongmentou), creating gradients in temperature, precipitation, and soil erosion. Coastal sites face salt spray, while upland areas experience cooler microclimates. Western arid corridor (e.g., Haiwei, Yinggehai, Yongming) is characterized by lower annual precipitation (<1200 mm), this region contrasts sharply with the island's humid eastern zones (e.g., Wene, Lianshan, Houhu, Lanyang) [31].

All samples collected from different sites were then planted into a randomized common garden design at Yangzhou University's experimental farm. This design ensures observed spectral variations primarily driven by genetic differences rather than environmental plasticity. Each sample was grown in an individual plot (0.25 m^2^ area) within a 500 m^2^ homogeneous field. The shortest distance between two neighboring plots was 1 m. Plants were grown in a standardized homogeneous loam soil (pH 6.8 ± 0.2) and received natural sunlight. All plots were managed with the same practices (daily irrigation, biweekly NPK fertilization) for one year prior to spectral measurement to ensure stable growth and trait expression.

### 2.2 Experimental design

Three datasets, natural leaf spectra, natural canopy spectra, and controlled leaf spectra (leaf spectra measured under controlled environmental conditions), acquired in three independent experiments under natural and controlled conditions, are summarized in the work flow in Fig. 1 and described in detail in the next sections. The three-spectral-dataset approach was designed to address distinct but interrelated research questions while controlling for potential confounding factors:

**Fig. 1:**
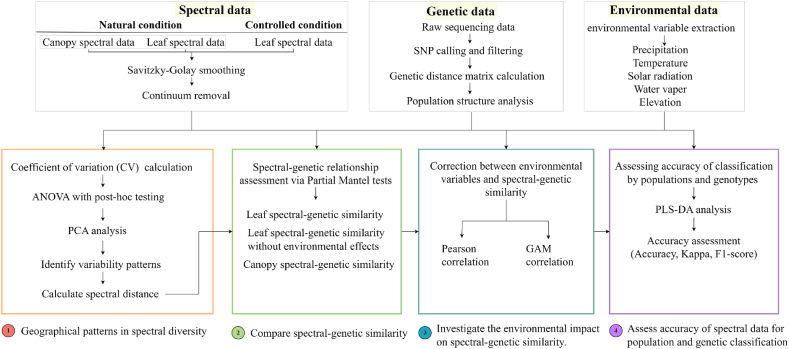
Framework of the study. Approaches towards linking spectral reflectance to genetic diversity.

Natural leaf spectra: Collected from leaves in their original growth environments, this dataset represents phenotypic expression under natural environmental heterogeneity. It addresses the primary research question: Can leaf reflectance spectra accurately distinguish genetically distinct populations in natural settings?

Natural canopy spectra: Acquired from canopy-level measurements in natural habitats, this dataset extends the investigation to the scale relevant for remote sensing applications. It addresses the research question: Can canopy reflectance spectra accurately distinguish genetically distinct populations in natural settings?

Controlled leaf spectra: Measured from the same populations grown in the controlled experimental field with homogeneous environmental conditions, this dataset isolates genetic effects on spectral traits by minimizing environmental variation. It addresses the research question: How much of the spectral variation observed in natural populations is attributable to genetic factors versus environmental plasticity?

The three datasets form an integrated analytical framework where each informs and validates the others: By comparing leaf- and canopy-level signals, we assess the scalability of spectral-genetic relationships; Comparison between natural and controlled leaf spectra quantifies environmental contributions to spectral variation; Consistency between leaf and canopy spectra supports the transferability of spectral signals across scales. Agreement across all three datasets strengthens the evidence for genetically determined spectral signatures. Based on the datasets, we conducted four major analyses to address our research questions (Fig. 1). The first analysis focused on investigating geographical patterns in spectral diversity across Hainan Island, mapping spatial variations in spectral reflectance to identify hotspots of phenotypic diversity in Bermudagrass. The second analysis aimed to compare spectral-genetic similarity, utilizing multivariate statistical approaches to evaluate correlations between spectral signatures and genetic variation. The third analysis explored environmental impacts on spectral-genetic similarity, integrating environmental variables to quantify how local abiotic conditions mediate the relationship between spectral and genetic data. Finally, the fourth analysis sought to assess the accuracy of spectral data for population and genetic classification, employing machine learning models to determine the predictive power of spectral reflectance in distinguishing genetically distinct populations.

### 2.3 Spectral data collection

Approximately 20 individual plants of bermudagrass per population were randomly chosen in each of the 14 geographic regions, with a distance of at least 20 m between neighboring plant sample locations. This random selection of individuals is intended for achieving representativeness of the bermudagrass population and reduces potential biases. Hyperspectral reflectance measurements were taken in situ on each individual sample at leaf and canopy level. After initial measurements under field conditions, leaf reflectance spectra were measured after plants had been acclimated in a common garden experiment for one year. We used a PSR + portable spectroradiometer (Spectral Evolution, Haverhill, MA, USA) equipped with a reflectance contact probe (featuring a tungsten halogen light source) and a leaf clip. The spectroradiometer covers a spectral range from 350 to 2500 nm, with a spectral resolution of 2.8 nm at 700 nm (FWHM), 8 nm at 1500 nm, and 6 nm at 2100 nm. **Leaf reflectance measurement**.

Leaf reflectance measurements were made at leaf adaxial surface, avoiding the main vein. A total of 15 measurements were taken per individual plant with 5 measurements for each of 3 distinct mats (clusters of leaves). The leaf clip was arranged to maximize the field of view by placing multiple narrow leaves across it, ensuring that the adaxial surface of the leaves faced the probe. This careful arrangement ensures that the leaf area covered in each measurement is consistent and representative. A minimum of 10 scans were taken for each leaf measurement. The mean reflectance spectrum was then calculated by averaging the spectral data for each individual plant. Prior to each measurement, a reference measurement was taken over a Spectralon® reference board. This step allows for spectral calibration using the reference measurement, ensuring consistency and accuracy across different samples under any fluctuations in light intensity or environmental conditions. After recording the white reference, the leaf clip was reversed to face a black background, allowing for dark current measurement.

### 2.4 Canopy reflectance measurement

Canopy spectral reflectance was also collected on the same individual plants as leaf reflectance measurements in the same wavelength range. Three spectral measurements were made per sample and a minimum of 10 scans were taken for each canopy measurement. The PSR + portable spectroradiometer was calibrated before each measurement to ensure accuracy. The spectroradiometer was mounted on a fixed-height, leveled boom to ensure consistent geometry across all measurements. The sensor was maintained at a nadir (0° zenith) orientation and a precise height of 35 cm above the canopy apex with a 25° field of view (FOV) for every measurement.

### 2.5 Genetic analysis

Fresh leaf samples were collected from 280 individuals across 14 geographic sites, with a minimum of 20 individuals genotyped per site. Samples were immediately preserved in silica gel for DNA extraction. Genomic DNA was extracted using the cetyltrimethylammonium bromide (CTAB) method [32], followed by quality assessment via spectrophotometry and agarose gel electrophoresis.

Single nucleotide polymorphisms (SNPs) were identified through SLAF-seq, a reduced-representation genome sequencing approach optimized for genetic diversity studies. SLAF paired-end libraries were constructed with the Nextera library kit following the protocol of Sun et al. (2013) [33] and sequenced on an Illumina HiSeq 2500 platform (Illumina, San Diego, CA, USA), generating 126-bp paired-end reads with a target depth of 40 × per genome.

Candidate SNPs were called using the GATK module SelectVariants. High-quality genome-wide SNPs were retained based on a minor allele frequency (MAF) > 0.05, and missing genotypes <50 %, following the SNP filtering criteria outlined by Yang et al. (2018) [34]. After filtering, 75,257 high-confidence SNPs were obtained, providing sufficient resolution for population-level genetic analyses.

To infer population genetic structure, we performed Bayesian clustering analysis using STRUCTURE v2.3.4 [35]. Ten independent runs were conducted for each *K* (1–20), with 100,000 Markov chain Monte Carlo (MCMC) iterations after a burn-in period of 50,000. The optimal number of genetic clusters (*K*) was determined by evaluating the Δ*K* statistic [36], which quantifies the rate of change in the log probability of the data [ln*P*(*D*)] between successive *K* values. A pronounced peak at *K* = 8 (Fig. S2; S3) indicated this as the most biologically meaningful partition. Beyond *K* = 8, the marginal gain in ln*P*(*D*) plateaued, suggesting no substantial improvement in explaining genetic variance with higher *K*.

### 2.6 Environmental data

Environmental data, including bioclimatic and topographical variables, was sourced from WorldClim at a 30 arc-second (∼1 km) resolution [37]. To accurately capture local environmental conditions, all variables were extracted as the mean value within a 1 km radius buffer surrounding each population's central GPS coordinate (WGS 1984), using the zonal statistics tool in QGIS v3.28 (QGIS Development Team 2023). This buffered approach accounts for microclimatic heterogeneity within the site's immediate vicinity.

The selected environmental variables were chosen based on their ecological relevance and potential impact on plant physiology, spectral reflectance, and genetic traits. We specifically focused on variables that are known to significantly influence plant growth and environmental adaptation, which include Annual mean temperature, Total annual precipitation, Elevation, Mean solar radiation, Mean water vapor content. Specificly, Annual mean temperature reflects the overall climate conditions, affecting metabolic rates, photosynthesis, and plant development; Total annual precipitation plays a key role in water availability, influencing plant moisture stress; Elevation affects temperature, precipitation patterns, and UV radiation exposure; Mean solar radiation provides insight into the energy available for photosynthesis; Mean water vapor content impacts photosynthesis and transpiration. The combination of these variables was selected to provide a well-rounded view of environmental factors likely to drive both spectral and genetic variation across the study sites.

### 2.7 Data analysis

#### 2.7.1 Spectral preprocessing

To ensure accuracy and consistency, the instrument was optimized before each sampling session and calibrated with a Spectralon® white reference panel every 5 min throughout data collection. Erroneous scans with reflectance values exceeding 1.0 were removed using R/SPECTROLAB [38]. We then applied Savitzky-Golay filtering to suppress high-frequency noise and performed continuum removal to isolate and normalize absorption features. Bands with strong atmospheric water vapor absorption were masked using the ENVI QUAC module for atmospheric correction. The leaf spectra were trimmed to the range of 400–2400 nm, excluding the high-noise regions between 350-399 nm and 2401–2500 nm from subsequent analysis, as suggested by Cavender-Bares et al. (2016) [39].

Canopy-level hyperspectral data were preprocessed to maximize the signal-to-noise ratio and mitigate confounding abiotic factors. We used canopy reflectance data in the 400–1300 nm wavelength range based on a quantitative signal-to-noise ratio (SNR) assessment. Furthermore, canopy spectra in the SWIR are highly susceptible to contamination from soil background features and residual atmospheric water effects, which act as non-genetic noise. While this excludes some SWIR-specific biochemical features, our selection prioritizes spectral stability and comparability across canopy samples, which is critical for robust downstream analysis.

### 2.8 Coefficient of variation and ANOVA analysis

The coefficient of variation (CV) was calculated to quantify the within-population variability in spectral data. Confidence intervals (95 %) were also estimated for CV values to assess the robustness of within-site variability patterns. A one-way Analysis of Variance (ANOVA) was performed to assess differences in mean spectral values among populations at various wavelengths. For each ANOVA test, P-values and effect sizes (eta-squared) were calculated to determine the statistical significance of spectral reflectance differences. All analyses and figure generation were conducted using the “dplyr”, “boot” packages in R v4.4.3, with “ggplot2” employed for visualization (version 4.4.3; R Core Team 2023).

### 2.9 Principal components analysis

To describe the spectral variance structure across populations, we performed principal component analysis (PCA) separately on the three spectral datasets obtained above using the “dplyr”, “factoextra” and “ggplot2” packages in R v4.4.3. For each dataset, we quantified the variance explained by each principal component (PC) and determined the proportion of total variance attributed to each. The number of PCs retained in the analysis was based on the cumulative explained variance, with components retained that explained at least 80 % of the total variance. This threshold is commonly used to balance data reduction with maintaining significant explanatory power in the analysis.

### 2.10 Spectral-genetic similarity estimation

For each of the three spectral datasets, we calculated spectral distance matrices using the Euclidean Distance to quantify dissimilarities in spectral profiles across populations. Nei's genetic distance matrix was derived from genetic data to assess genetic divergence among populations, while Spatial Euclidean Distance was calculated based on the geographical position of the populations.

To evaluate spectral-genetic relationships, we performed the Partial Mantel Tests [40,41], to correlate each spectral distance matrix with the genetic distance matrix while controlling for spatial distance. Partial Mantel correlations were calculated using the mantel.partial function in the vegan package in R v4.4.3. The statistical significance of the partial correlation coefficient (r) was assessed using 9999 permutations to generate a robust P-value. Given that tests were performed across 2000 spectral bands, we applied a False Discovery Rate (FDR) correction (Benjamini-Hochberg procedure) to the resulting P-values to control for Type I errors arising from multiple comparisons. Prior to the analysis, potential multicollinearity between predictors (genetic and spatial distances) was evaluated by Pairwise Mantel Tests between genetic and spatial distances, allowing for a clearer interpretation of the relationships between spectral reflectance, genetic distance, and environmental factors.

To investigate the extent to which variation in spectral-genetic similarities among the 14 bermudagrass populations is explained by environmental variables at different geographic sites, we calculated the Pearson correlations between spectral-genetic similarities and environmental variables. To complement these linear analyses, we also applied generalized additive models (GAMs), which revealed non-linear relationships between spectral-genetic similarity and certain environmental variables. The analysis was performed for each dataset under natural conditions to evaluate the effects of environmental variables, such as temperature, precipitation, solar radiation, water vaper, and elevation, on spectral-genetic similarity.

### 2.11 PLS-DA analysis

Populations and genotypes were classified using the partial least squares discriminant analysis (PLS-DA), a multivariate method that classifies observations based on PLS regression of indicator variables [42]. The PLS-DA procedure was conducted separately for natural leaf and canopy spectra among populations, utilizing a random 80 % of the spectral data as a training set and the remaining 20 % as the testing set for the classification model. To ensure the robustness and generalizability of the model, we employed a rigorous repeated k-fold cross-validation scheme. Specifically, we used 10-fold cross-validation with 5 repeats. Model performance was then evaluated using a comprehensive set of metrics (Overall accuracy and Cohen's kappa and F1-scores) calculated from the aggregated predictions across all 50 resampling iterations. Additionally, the same PLS-DA analysis was applied to classify natural leaf spectra into different genotypes, assessing the potential of leaf spectral data to estimate genetic variation under different environmental conditions.

We used the ‘vip’ function from the mixOmics package (v6.18.1) with default parameters to calculate VIP scores were from our PLS-DA models. To assess statistical significance, we performed permutation tests (999 permutations) where class labels were randomly shuffled, and VIP scores were recalculated.

### 2.12 Spatial autocorrelation analysis

To quantify the degree of spatial autocorrelation in our spectral data, we conducted Moran's I analysis on principal components derived from natural leaf and canopy reflectance spectra. Moran's I indices were calculated using the ‘moran.test()‘ function from the R package ‘spdep’, with statistical significance assessed through 999 permutation tests. Moran's I ranges from −1 (perfect dispersion) to +1 (perfect clustering), with 0 indicating no spatial autocorrelation.

## 3 Results

### 3.1 Spectral variance across populations

Our multi-dataset approach—encompassing natural leaf, controlled leaf, and natural canopy spectra—reveals consistent population-level spectral differentiation while quantifying the influence of environmental and measurement-scale effects. The average spectral reflectance for each population across different wavelengths is plotted to visually compare spectral characteristics (Fig. 2a–c). For natural leaf spectra, populations exhibited distinct reflectance profiles, with particularly pronounced differences in the NIR and SWIR regions (Fig. 2a). Spectral differences among populations could reflect variations in leaf structure, moisture content, or chemical composition, highlighting the potential of spectral reflectance for differentiating populations and assessing optical properties. These patterns were preserved and even enhanced in the controlled leaf dataset (Fig. 2b), indicating a strong genetic basis for the observed spectral signatures, as environmental variability was minimized. Canopy-level spectra largely mirrored leaf-level trends but with amplified population distinctions in the NIR region, demonstrating the scalability of these spectral-genetic relationships (Fig. 2c).

**Fig. 2:**
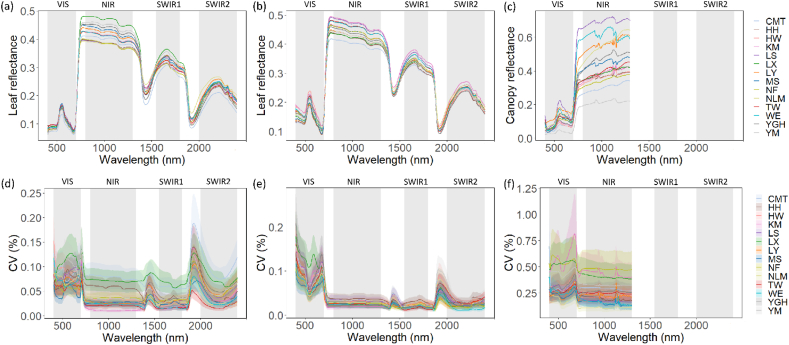
Within-population spectral variability across datasets. (a–c) Mean reflectance spectra: (a) natural leaf, (b) controlled leaf, and (c) natural canopy datasets. (d–f) coefficient of variation (CV) with shaded areas indicating 95 % confidence intervals: (d) natural leaf, (e) controlled leaf, and (f) natural canopy datasets.

Fig. 2d and e illustrate the CV in leaf reflectance across populations. The greatest variability was detected spectral variation within population. The result suggests that certain populations under natural conditions exhibit more heterogeneous reflectance properties in these spectral regions compared with leaf spectral measurements under controlled conditions. In contrast, Fig. 2f shows CV of canopy reflectance spectra, revealing that CV of canopy spectral reflectance is generally higher than that of leaf spectral reflectance. The broader confidence intervals for canopy CV highlight greater uncertainty, largely attributable to canopy structural complexity (e.g., leaf angle distribution and shadowing effects). The ANOVA analyses indicated significant spectral differences among populations across most wavelengths (P < 0.05) for natural leaf, controlled leaf and natural canopy data (Fig. S4).

The results of the PCA analysis of spectral data across different populations are presented in Fig. 3, along with the contribution of various spectral wavelengths to the first two principal components (PCs). In Fig. 3a, PCA results for the first two principal components (PC1 and PC2) are shown, with PC1 accounting for 47.0 % of the variance and PC2 explaining 35.8 % of the variance. The scatter plot shows that certain populations exhibit greater spectral variability than others. For example, populations “CMT” and “HH” are more dispersed along both principal components, indicating higher spectral diversity, whereas “TW” and “YM” form more compact clusters. Fig. 3b displays PCA results for the controlled leaf spectral dataset, where PC1 and PC2 explain 59.3 % and 23.3 % of the variance, respectively. A clearer separation along PC1 is observed, with populations like “CMT” and “NF” positioned further apart from others. PCA analysis of leaf spectra under both natural and controlled conditions showed clear population clustering (Fig. 3a and b), with genetic effects explaining a greater proportion of variance when environmental noise was removed. In contrast, canopy-level PCA was dominated by a single principal component (PC1 explaining 89.8 % of variance; Fig. 3c). The clustering patterns are more tightly concentrated around the origin for most populations compared with the leaf spectral datasets, suggesting reduced spectral variability at the canopy level under natural conditions.

**Fig. 3:**
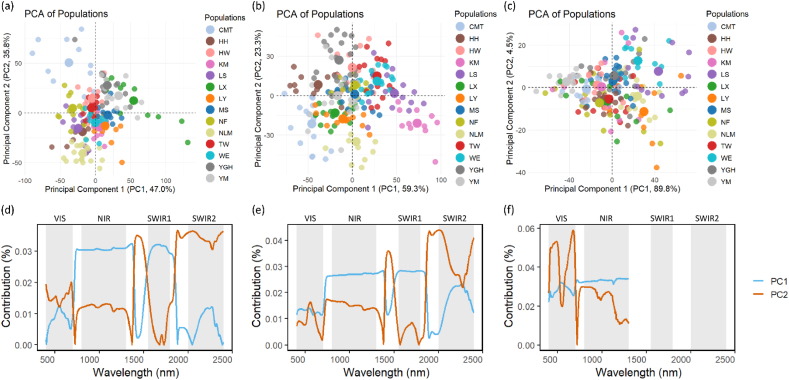
Results from Principal component analysis (PCA) of spectral data across different populations. (a–c) PCA score plots in the space of the first two components: (a) natural leaf spectra, (b) controlled leaf spectra, and (c) natural canopy spectra. (d–f) wavelength contributions to PCs for: (d) natural leaf spectra, (e) controlled leaf spectra, and (f) natural canopy spectra.

For leaf spectral reflectance, the NIR and SWIR regions significantly contribution to both PCs, with prominent peaks at key absorption bands (e.g., water absorption at ∼1450 nm and ∼1940 nm) and regions of high reflectance (Fig. 3d and e). PC1 (blue line) exhibits a more evenly distribution of contributions across the spectrum at canopy level, while PC2 (orange line) shows sharper peaks aligned with specific spectral features in VIS region (Fig. 3f). This suggests that PC2 captures finer-scale spectral variability related to water content or other plant biochemical properties.

### 3.2 Spectral-genetic similarity assessment

Mantel test showed a non-significant negative correlation between genetic and geographic distances (r = −0.218, p = 0.867). This result suggests minimal multicollinearity between these predictors, validating our use of partial Mantel tests. Spectral-genetic similarity, quantified by the partial Mantel correlation between spectral distance and genetic distance, ranged from 0 (low similarity) to 0.7 (high similarity) across wavelengths. The overall patterns are summarized in the correlation r value heatmap (Fig. 4), while the corresponding P-values are provided in Supplementary Fig. S5. For natural leaf spectra, significant correlation (r ranges from 0.4 to 0.7; P < 0.05; Fig. 4a–S5) were detected in the shortwave infrared (SWIR) region between 1900 and 2400 nm for natural leaf dataset, with the exception of a narrow window between 2100 and 2300 nm. Spectral-genetic similarity over the SWIR regions reached its highest values. This pattern persisted but became more focused in the controlled leaf spectra, where high correlations (r = 0.4–0.6) were concentrated in the 1900–2000 nm range (Fig. 4b–S5). The consistency across both leaf datasets confirms that SWIR reflectance, linked to leaf water content and structural biochemistry, carries a robust, heritable signal. In contrast, canopy spectra showed negligible spectral-genetic correlations across the VIS and NIR regions, demonstrating that genetic signals are attenuated when scaling from leaf to canopy, likely due to structural complexity and background interference (Fig. 4c).

**Fig. 4:**
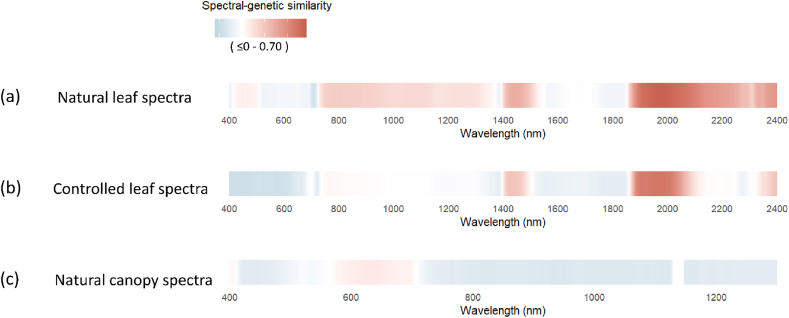
Spectral-genetic similarities (values of the partial Mantel r) derived from spectral bands for each dataset. (a) natural leaf spectral dataset, (b) controlled leaf spectral dataset, and (c) natural canopy spectral dataset.

The relationship between spectral-genetic similarity and environmental factors differed markedly between measurement scales. Results showed correlations between various environmental factors and spectral-genetic similarity using Pearson's correlation across wavelengths from 400 nm to 2400 nm for both natural leaf and canopy spectral dataset (Fig. 5a). Leaf spectra-genetic similarity showed significant liner correlations with these environmental variables in the SWIR region (r > 0.4; P < 0.05; Fig. S6), consistent with physiological processes such as leaf water content and drought responses. Generalized additive models (GAMs) analysis revealed significant non-linear correlation between leaf spectral-genetic similarity and precipitation in the SWIR bands (Fig. 6a; S7).

**Fig. 5:**
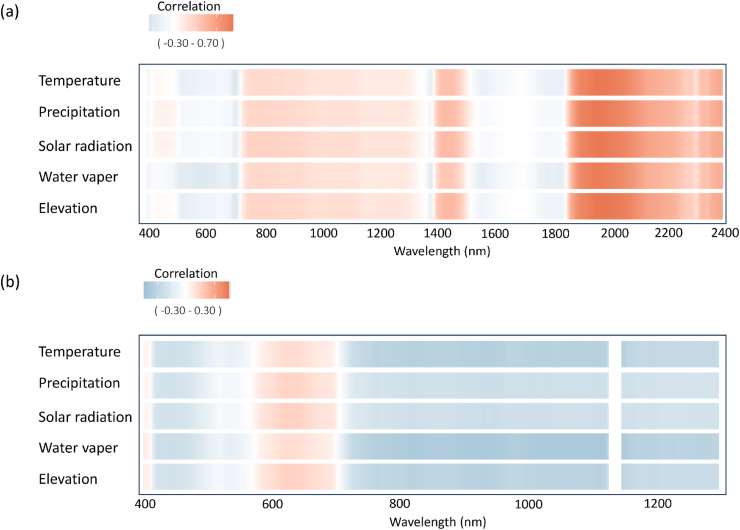
Pearson correlations between spectral-genetic similarities and environmental variables at each spectral band. (a) natural leaf spectral dataset, and (b) natural canopy spectral dataset.

**Fig. 6:**
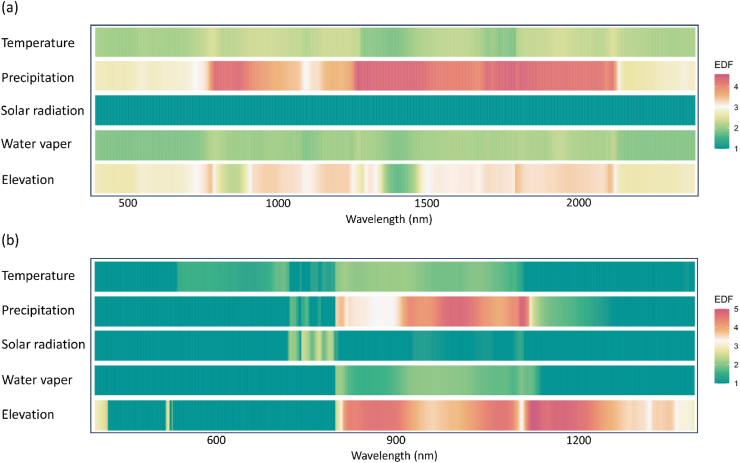
GCM correlations between spectral-genetic similarities and environmental variables at each spectral band. (a) natural leaf spectral dataset, (b) natural canopy spectral dataset.

Canopy spectral-genetic similarity shows a narrower range of liner correlation values (−0.30 to 0.30) (Fig. 5b; S8), with generally weaker correlation than those observed in leaf spectra and fewer pronounced regions of high correlation. However, canopy spectral-genetic similarity presented significant non-liner correlations with precipitation and elevation in the NIR regions (800–1200 nm; EDF >3; P < 0.05; Fig. 6b; S9). Canopy hyperspectral measurements typically bring additional information due to factors such as multiple scattering effects, structural complexity, background interference, and environmental variability. These sources of external information can reduce the strength of correlations relative to leaf spectra.

### 3.3 PLS-DA model performance

PLS-DA achieved high accuracy in classifying populations using natural leaf spectra (Accuracy = 86.76 %; Kappa = 0.86) compared to canopy data (Accuracy = 41.74 %; Kappa = 0.37) (Fig. 7a and b; Table S2), confirming that leaf-level measurements minimize confounding structural and environmental noise. Leaf spectral data (Fig. 7c) also performed high classification accuracy (Accuracy = 83.32 %; Kappa = 0.81), depending on the genetic clusters across the populations. These high accuracy rates suggest that leaf spectral reflectance captures distinctive features closely linked to genetic or phenotypic differences among the populations. Notably, the pattern of misclassification in the confusion matrix is ecologically informative rather than random. Most misclassifications occurred between geographically proximate populations with similar environmental conditions, such as between the Nanfeng (NF) and Wene (WE) sites (Fig. S1).

**Fig. 7:**
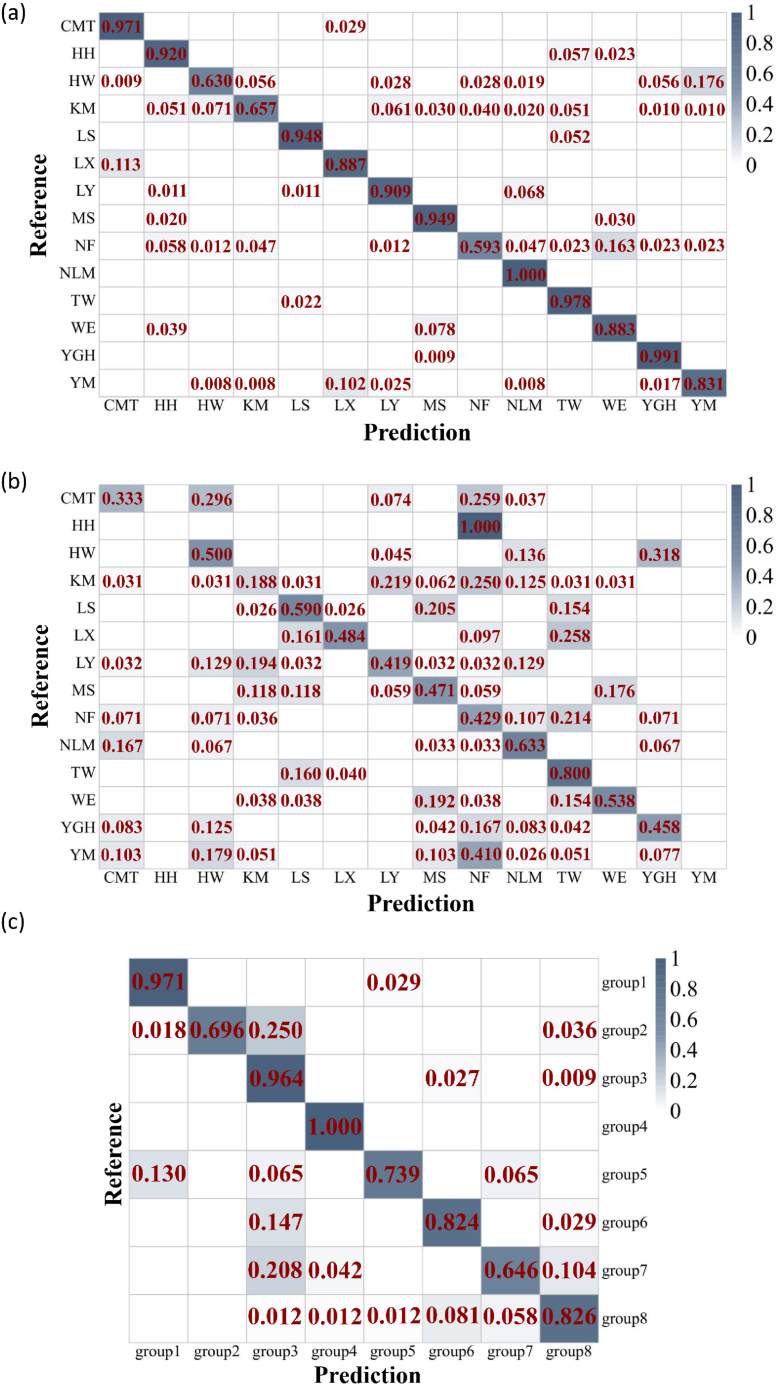
Confusion matrix from partial least square discriminant analysis (PLS-DA) classification. (a) population classification using natural leaf reflectance spectroscopy, (b) population classification using natural canopy spectral data, and (c) genotype classification using natural leaf reflectance spectroscopy.

Although standard cross-validation indicated high classification accuracy for leaf spectra, we conducted Variable Importance in Projection (VIP) and Moran's I analysis to evaluate model robustness and mitigate concerns about potential overfitting due to spatial autocorrelation or high-dimensional data. We compared the full-spectrum model against a model built using only features selected by Variable Importance in Projection (VIP). The VIP analysis identified 111 spectral bands representing genuine biological signal (Fig. S10), which are considered the most relevant for discriminating populations. The PLS-DA model for all three datasets trained solely on these VIP-selected bands yielded a lower classification accuracy than full-spectrum model (Table S2; Accuracy = 72.1 %; Kappa = 0.70 for natural leaf spectral data). The results demonstrate that while a subset of wavelengths carries strong discriminatory power, the full spectral model leverages synergistic information across a broader range of wavelengths. The superior performance of the full-spectrum model indicates that its high accuracy is not an artifact of overfitting but is instead driven by the integration of robust, complementary signals pervasive throughout the spectral reflectance profile. Moran's I analysis revealed statistically significant but practically negligible spatial autocorrelation in spectral data. For natural leaf spectral data, global Moran's I was −0.003 (p < 0.001), while canopy spectral data exhibited Moran's I of 0.002 (p < 0.001). These near-zero values indicate that spatial proximity among samples contributes minimally to observed spectral variation. Spatial autocorrelation analysis revealed minimal spatial structuring in the spectral data.

## 4 Discussion

### 4.1 Geographic patterns of spectral diversity across Hainan island related to genetic variation

Our findings underscore the considerable spectral variability within populations and the significant spectral differences observed across populations, highlighting the importance of geographic site-specific factors in shaping the optical properties of leaves and canopies in the NIR and SWIR regions. These steep environmental gradients on Hainan Island drive local adaptations in leaf morphology and biochemistry—such as variations in leaf mass per area (LMA) and non-structural carbohydrate content—which are strongly captured by SWIR reflectance [43]. Reflectance spectra provide valuable insights across multiple levels of biological diversity, extending beyond the individual level [20]. Prior studies have linked leaf reflectance to biochemical traits such as chlorophyll and water content, demonstrating the impact of geographic variation on these traits [44]. Compared to controlled settings, natural leaf spectral variance increased in SWIR and NIR wavelength, highlighting the role of environmental heterogeneity in amplifying phenotypic diversity. The observed spectra differences suggest local adaptations to Hainan's unique bioclimatic conditions, likely reflecting variations in leaf morphology, moisture content, and plant biochemical composition driven by drought stress and high solar irradiance—key challenges for tropical grasses under climate change [45].

While partial Mantel tests provide a valuable framework for assessing the relationship between spectral and genetic distances while controlling for a third variable (geographic distance), we acknowledge the limitation that partial Mantel tests assume linear relationships and may not fully account for complex, non-linear spatial dependencies or unmeasured environmental covariates that co-vary with geography. However, the specific conditions of our system mitigate several of these concerns. The very weak and non-significant correlation we found between genetic and geographic distances (Mantel r = −0.218, p = 0.867) means that multicollinearity—a major source of bias in partial regression techniques—is minimal. This strengthens our interpretation that the strong spectral-genetic correlation in the shortwave infrared (SWIR, 1900–2400 nm) represents a genuine biological relationship rather than a statistical artifact. On a theoretical level, the prominence of the SWIR region can be interpreted through plant optical principles [46]. SWIR reflectance primarily captures internal chemical pools and dry matter properties, which are more evolutionarily conserved, more directly influenced by genetic architecture, and less susceptible to short-term environmental fluctuations compared to VIS/NIR traits governed by the strong absorption of radiation by leaf pigments. Physiologically, the SWIR reflectance (1900–2400 nm) is predominantly more sensitive to leaf compounds such as water [47] and biochemical (e.g. nitrogen, protein, lignin, and cellulose) [48,49] and phenolic compounds [50]. The most pronounced features are water absorption within a leaf near 1950, and 2500 nm [51]. Spectral features are tied to molecular bond vibrations (e.g., C-H, N-H, O-H) characteristic of lignin, cellulose, and nitrogen [52]. The widespread and significant correlations here imply that leaf carbon and nitrogen economy spectra (e.g., defense compounds, structural investment) may have a heritable component expressed in the reflectance signature. This inference is supported by our controlled-environment dataset results, where the correlation peak in this region became narrower and more distinct, indirectly supporting the link between this band and conserved chemical properties. The strong correlations in the 1900–2000 nm range, which encompasses a major water absorption feature (∼1940 nm) and overlapping bands for cellulose and lignin, suggest that genetically influenced variation in water-use strategy, cell wall architecture, and bulk carbon allocation are key drivers of spectral diversity in our system [53]. This suggests that reducing environmental noise allows the intrinsic spectral signals related to conserved genetic factors—potentially linked to water and core chemistry—to be more clearly resolved. It is important to note that this study represents an exploratory analysis based on correlations between spectral and genetic distances. We demonstrate a robust spectral-genetic association in the SWIR and propose its potential trait basis grounded in optical theory. However, to establish definitive causal links, future research must integrate triadic measurements of “spectra-traits-genotypes."

A comparative analysis of the natural and the controlled datasets allows us to explore the relationship between spectral traits and genetic diversity in bermudagrass. We observed significant spectral-genetic similarities in the natural leaf reflectance dataset across a broader range of wavelengths, particularly in the SWIR regions, when compared with the controlled leaf spectra. This underscores that in-situ spectra capture the expressed phenotypic plasticity under real-world selection pressures, providing a more holistic measure of a population's adaptive capacity [54]. Our findings reveal that spectral variation in bermudagrass populations arises from the interplay of genetic divergence and environmental adaptation, mediated by physiological traits encoded in key spectral regions. On Hainan Island, this interplay is intensified by steep environmental gradients. For example, elevated SWIR reflectance in populations in arid-region correlates with genetically driven traits such as leaf cuticle thickness and reduced stomatal density [55]. Conversely, populations from humid margins exhibited higher NIR reflectance, a plastic response to optimize light capture in shaded understories through altered leaf angles, a phenotype modulated by both phytochrome gene expression and microenvironmental light gradients [56]. By integrating spectral data with genetic information, we can examine whether variations in spectral characteristics are linked to genetic diversity, thereby offering insights into how populations adapt to their environments. Our approach aligns with oak [39] and cottonwood (*Populus* spp) [57] community spectroscopy but diverges by focusing on intra-specific variation within a tropical grass system, where spectral variation was driven by Island habitat due to higher environmental heterogeneity.

However, several limitations of the common garden approach must be acknowledged, particularly for perennial species like bermudagrass. A key consideration is the potential for carryover effects—where traits developed in the original field environments (e.g., leaf structural and biochemical properties such as cell wall composition or leaf mass per area) persist during the acclimation period. While the common garden effectively standardizes current above-ground conditions, it may not fully reset these established physiological states, especially in perennial grasses where such adjustments can be gradual. We explicitly recognize that our standard acclimation period may not have been sufficient to completely erase all prior environmental imprints on plant phenotype. Consequently, the spectral-genetic correlations we observed, particularly in the SWIR region which is sensitive to persistent structural and chemical traits, likely reflect a composite signal of constitutive genetic differences and environmentally mediated, yet persistent, phenotypic adjustments. Additionally, although we minimized microenvironmental variation, subtle gradients in temperature and light exposure across the garden may interact with genetic backgrounds, partially masking genotype-environment interactions that would be fully expressed in natural settings. Future studies employing longer acclimation phases, monitoring trait ontogeny, or implementing reciprocal transplant experiments across environmental gradients would be valuable to better disentangle genetic and plastic components of spectral variation.

While leaf reflectance retained strong spectral-genetic correlations, canopy-level spectra exhibited markedly weaker associations with genetic diversity under natural condition. For example, the weak spectral-genetic correlations observed at the canopy level in natural conditions suggest that genetic variation is less pronounced in canopy reflectance compared to leaf reflectance [58]. However, this attenuation of the genetic signal is not merely due to increased noise [59] but can be systematically interpreted through the principles of canopy radiative transfer. The canopy reflectance is an integrated signal where the leaf biochemical properties (carrying genetic information) are convoluted with several confounding physical and structural factors: (1) Canopy architecture—including leaf area index (LAI), leaf angle distribution, and clumping—governs light interception and multiple scattering. These structural traits exhibit high phenotypic plasticity in response to competition, density, and micro-environmental gradients [60]. This plasticity introduces substantial spectral variance that is not directly genetically coded, effectively masking the more subtle biochemical spectral differences linked to genotype. (2) The spectral signal from non-photosynthetic elements, such as soil, litter, senesced material, and stems, forms a variable background. In sparse or heterogeneous canopies, this background contribution can be dominant and spectrally distinct from green vegetation, diluting the relative spectral influence of living leaves where genetic information is primarily expressed. (3) Canopy reflectance is inherently anisotropic, governed by the Bidirectional Reflectance Distribution Function (BRDF). Variations in solar zenith angle, view angle, and relative azimuth cause significant changes in measured reflectance due to shadowing and illuminated component fractions. These geometry-driven variations, which change diurnally and seasonally, are largely independent of genetic variation and can obscure genotype-specific spectral signatures. Therefore, the weak spectral-genetic correlations observed at the canopy level likely represent a significant underestimation. We explicitly acknowledge that a key limitation of this study is the lack of quantitative correction for these effects. Overcoming this integration challenge is essential for scaling spectral genetics to landscape monitoring. Promising paths forward include: (1) using radiative transfer models (RTMs) like PROSAIL in an inverse mode to separate leaf biochemical from canopy structural contributions; (2) fusing hyperspectral data with LiDAR or structure-from-motion data to explicitly account for 3D canopy architecture; and (3) employing multi-temporal observation strategies to normalize for illumination and phenological states [61,62].

### 4.2 Environmental modulation of genetic signals in spectral data

This observation supports the concept of genotype-by-environment interactions, where the expression of genetic traits, such as leaf reflectance and photosynthetic activity, is strongly shaped by local environmental conditions [63]. These results underscore the necessity of contextualizing spectral-genetic relationships with environmental covariates to avoid conflating plasticity with heritable divergence.

The correlation between leaf spectral-genetic similarities and key environmental variables, such as temperature, precipitation, and solar radiation, was particularly strong in the SWIR regions. This spectral range is highly sensitive to plant hydration and leaf structure, both of which are influenced by environmental conditions [64]. The strong correlation aligns with previous researches showing that water absorption features in the SWIR region are closely linked to variations in plant water content, which is shaped by both genetic and environmental factors [[65], [66]]. In this context, genetic traits influencing water retention, such as cuticle thickness and stomatal conductance, become crucial for understanding how spectral signatures reflect both genetic adaptation and environmental responses [26]. Canopy spectral-genetic similarities presented significant non-liner correlation with precipitation and elevation in the NIR spectra, suggesting that environmental influences on canopy reflectance may be more complex or indirect.

However, it is important to acknowledge a key methodological limitation: our environmental variables were primarily derived from global-scale climate databases (WorldClim) at 30-arc second (∼1 km) resolution. While these data adequately capture broad regional gradients across Hainan Island, they may not fully resolve micro-environmental variation at individual plant level that could further refine spectral-genetic relationships. Ideally, in-situ measurements of soil properties and microclimate conditions would provide a more mechanistic understanding of the drivers of spectral and genetic variation [67]. Linking environmental factors to genetic variation through remote sensing provides valuable insights into the adaptive processes driving biodiversity patterns and the conservation of genetic resources [68,69]. These findings emphasize the importance of accounting for local environmental conditions in future studies of plant spectral variation and genetic diversity, particularly through the integration of directly measured site-level variables alongside remotely-sensed climate products.

### 4.3 Potential of spectral reflectance in distinguishing different populations and genotypes in situ

Our study demonstrates that leaf reflectance spectroscopy effectively captures sufficient information to distinguish among the sampled bermudagrass populations and genotypes, as evidenced by the high classification accuracy of the PLS-DA model. However, the interpretation of this high accuracy requires careful consideration. Our additional analyses directly address this concern: Moran's I analysis revealed statistically significant but practically negligible spatial structuring in spectral data (I ≈ 0.002), indicating that spatial proximity contributes minimally to spectral variation. The performance advantage of full-spectrum models over VIP-selected models further demonstrates that models utilize complementary information distributed across the spectral range rather than overfitting to a limited subset of bands. Our cross-validation approach aimed to mitigate overfitting, but we acknowledge that the model's generalizability to entirely independent populations from unsampled regions remains untested and constitutes a key limitation for operational application. Therefore, the primary conclusion from our classification analysis is not that we have built a universally applicable discriminator, but that the spectral differences between these a priori defined groups are strong and systematic enough to allow their separation within this dataset. This separability has ecological meaning: populations from environmentally divergent regions (e.g., arid coastal vs. humid inland sites) were rarely confused in the confusion matrix, underscoring how strong environmental selection can drive correlated spectral and genetic divergence. Notably, the few observed misclassifications in the PLS-DA provide valuable insights, potentially indicating gene flow between nearby populations or convergent adaptation to similar local habitats, leading to a shared spectral signature. This finding aligns with previous research using aerial imaging spectroscopy to distinguish Geographical origin discrimination of lemon myrtle (*Backhousia citriodora*) [70]. NIR and SWIR features associated with leaf pigments, lignin, cellulose, and phenolic compound contents provided the strongest discriminatory power, reflecting geographic variation in biosynthetic pathways [71]. Our finding reinforces the potential of leaf spectral data for detecting fine-scale genetic diversity in situ, supporting the idea that reflectance spectral data convey evolutionary relevance [[72], [73], [74]]. Previous studies have demonstrated that spectral data effectively capture genetic variation related to leaf structure, biochemical composition, and water content [75,76].

To translate this potential into a robust tool, future work must prioritize validation frameworks that directly address this generalizability limitation. This includes: (1) External validation using spectrally and genetically characterized populations from completely independent geographic regions not used in model training; and (2) employing spatial blocking or k-fold cross-validation schemes explicitly designed to ensure that spatially proximate samples are not split across training and testing sets in a way that artificially inflates accuracy. Such steps are essential to confirm that the spectral signals learned are truly indicative of genetic variation rather than local environmental covariance or spatial autocorrelation.

## 5 Conclusion

This geographic pattern of spectral variation was especially pronounced along Hainan's steep environmental gradients, where populations from different sites showed distinct NIR and SWIR signatures linked to genetically adapted traits. Spectral-genetic correlations (r = 0.4–0.7; P < 0.05) were significant in natural environments in the SWIR regions (1900–2400 nm), where environmental heterogeneity amplifies phenotypic diversity through genotype-by-environment interactions. Canopy-level spectral data showed markedly reduced sensitivity to genetic diversity (P > 0.05), highlighting the challenges posed by structural complexity and environmental buffering effects in field conditions. The enhanced genetic resolution of leaf spectral data, compared to in situ canopy measurements, reflects phenotypic adaptations of foliar traits to heterogeneous environmental gradients across Hainan Island. Significant liner correlation (r > 0.4, p < 0.05) between environmental drivers and leaf spectral-genetic similarity revealed that we disentangled SWIR bands as heritable spectral signals from plastic phenotypic responses.

Leaf-level spectroscopy serves as a powerful proxy for detecting genetic differences, with our PLS-DA model achieving high accuracy in classifying populations spectra (Accuracy = 86.76 %; Kappa = 0.86) and genetic clusters (Accuracy = 83.32 %; Kappa = 0.81) based solely on spectral signatures. This high classification performance, maintained even under heterogeneous field conditions, directly confirms that leaf spectral data contain strong genetic signals. This study establishes leaf reflectance spectroscopy as a scalable tool for in situ genetic diversity monitoring, with direct applications in conservation and precision breeding. By prioritizing SWIR/NIR bands, our framework allows for more accurate and quick mapping of adaptive functional variation under genetic selection, providing potential insights for management and restoration in ecosystems where genetic resilience is most urgently needed. By bridging spectral phenomics and landscape genomics, this framework offers a scalable approach to conserve genetic resources and optimize plant resilience in a changing climate.

## CRediT authorship contribution statement

Jingxue Zhang: Writing – review & editing, Writing – original draft, Investigation, Formal analysis, Data curation, Conceptualization. Jiali Shang: Writing – review & editing, Software, Methodology. Jiangui Liu: Writing – review & editing, Software, Methodology. Liwen Wang: Investigation. Mengli Han: Investigation. Minghui Chen: Investigation. Chen Chen: Investigation. Yuhong He: Writing – review & editing, Supervision, Software, Methodology. Xuebing Yan: Supervision, Conceptualization, Writing – review & editing, Funding acquisition.

## Ethics statement

This research complied with China's Regulations on Access to Biological Resources. Specimen collection was authorized by Hainan Provincial Authorities and Yangzhou University Ethical Committee.

## Data availability statement

We have deposited SNP data in Dryad. Data available from the Dryad Digital Repository:

https://doi.org/10.5061/dryad.vdncjsz4z.

Reviewer sharing link: http://datadryad.org/stash/share/7iJJ2CYijIxzNviUlCdPQm4ENX4G7OR_ERArLN1mZfo.

## Funding statement

Financing was granted by 10.13039/501100001809National Natural Science Foundation of China (32171672).

## Conflict of interest

The authors declare that they have no known competing financial interests or personal relationships that could have appeared to influence the work reported in this paper.

## Figures and Tables

**figs1:**
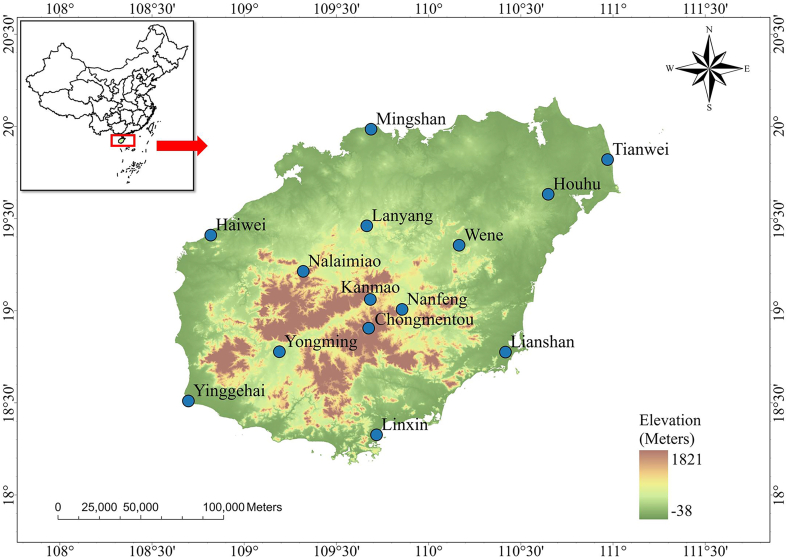


**figs2:**
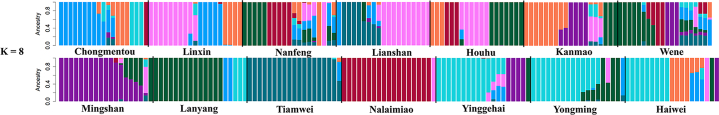


**figs3:**
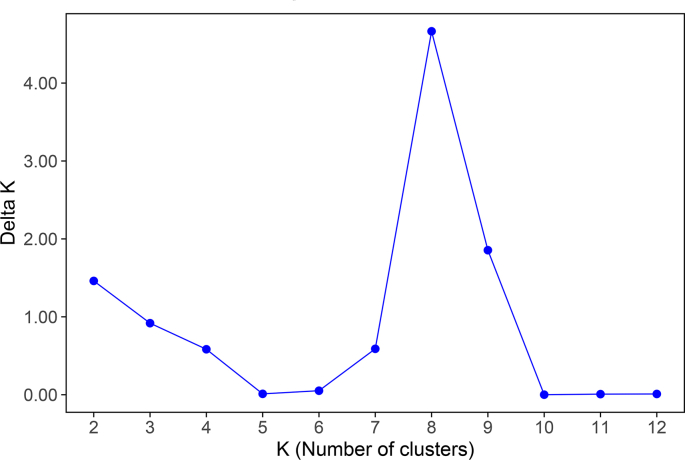


**figs4:**
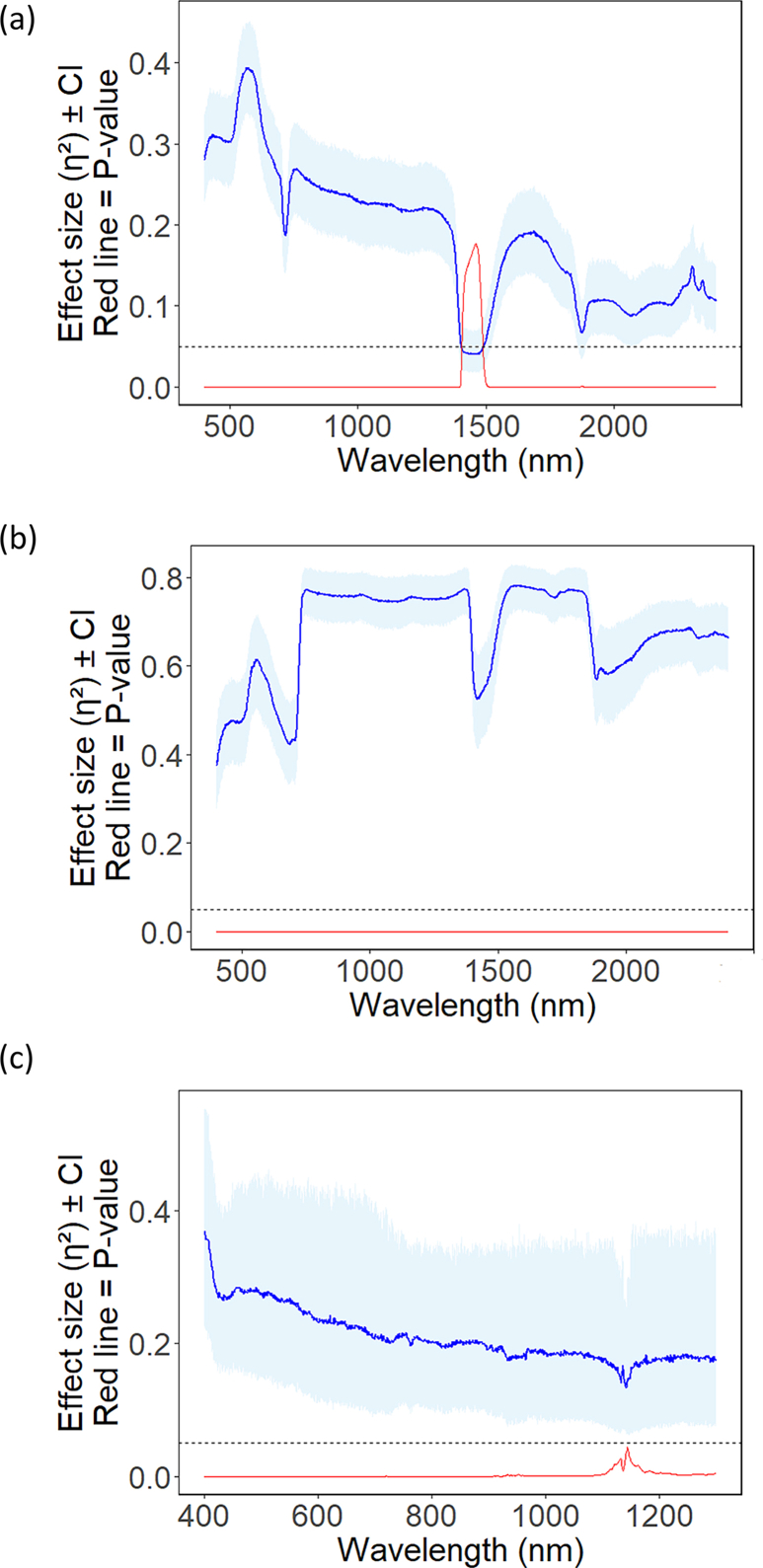


**figs5:**
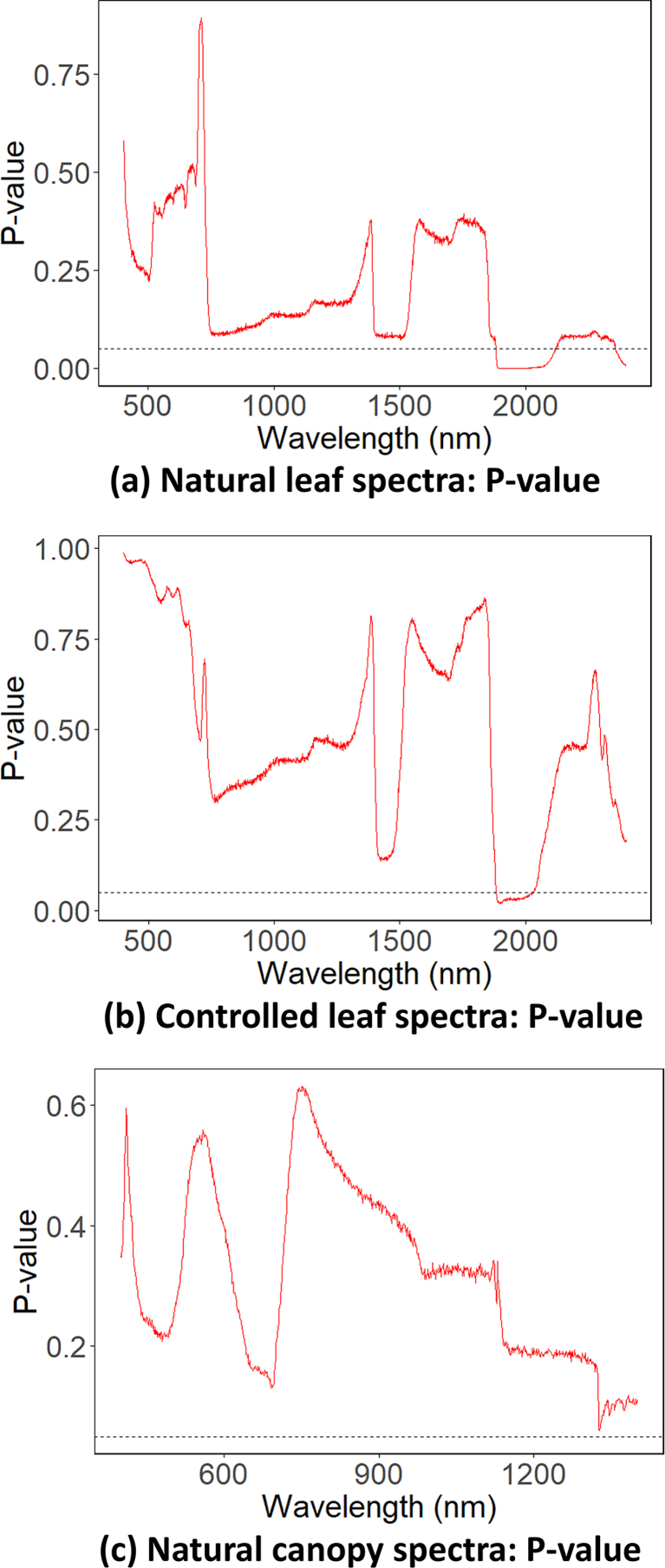


**figs6:**
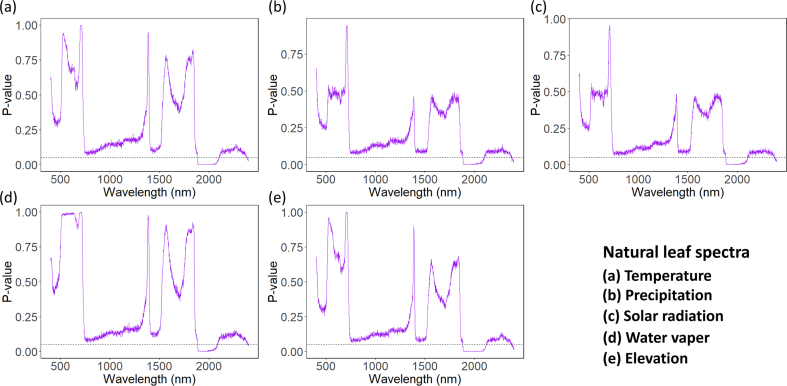


**figs7:**
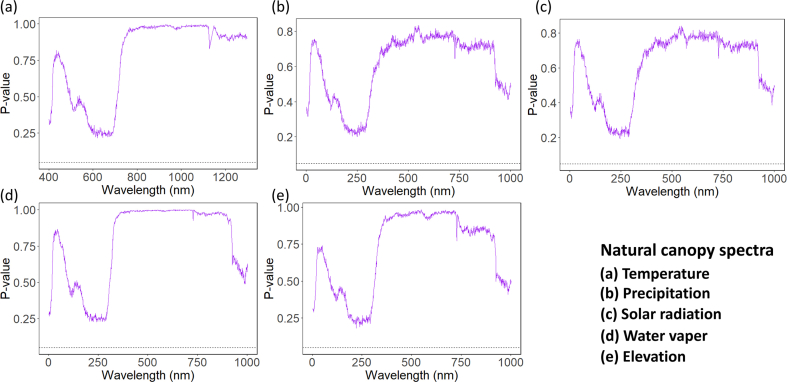


**figs8:**
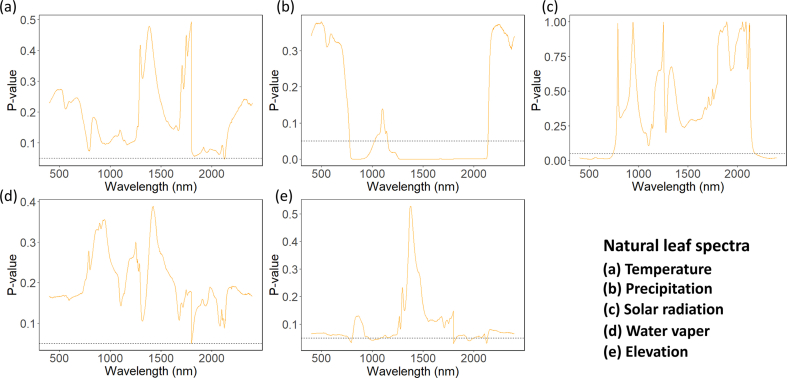


**figs9:**