# Supplementary material for: Genetic constraints on in-situ reflectance spectral variation in bermudagrass populations across Hainan Island
Source: Plant Phenomics. 2026 Jan 14;8(1):100168. doi: 10.1016/j.plaphe.2026.100168 (PMC13109577; doi:10.1016/j.plaphe.2026.100168)
Supplement: Multimedia component 1 [file mmc1.docx]

| **Locations** | **Latitude** | **Longitude** | **Ns** | **Elevation (m)** | **Average solar radiation** | **Average water vaper** | **Average temperature (°C)** | **Average precipitation (mm)** |
| --- | --- | --- | --- | --- | --- | --- | --- | --- |
| Mingshan | 19.98°N | 109.69°E | 20 | 12.68 | 17204.59 | 2.47 | 24.18 | 134.53 |
| Lanyang | 19.46°N | 109.66°E | 20 | 197.82 | 17273.77 | 2.39 | 23.80 | 144.66 |
| Chongmentou | 18.90°N | 109.67°E | 20 | 665.56 | 17224.44 | 2.18 | 21.72 | 137.09 |
| Linxin | 18.33°N | 109.72°E | 20 | 1.46 | 17916.22 | 2.62 | 25.73 | 122.10 |
| Haiwei | 19.41°N | 108.82°E | 20 | 26.10 | 17221.93 | 2.49 | 24.79 | 103.81 |
| Nalaimiao | 19.21°N | 109.32°E | 20 | 316.68 | 17380.95 | 2.32 | 23.26 | 124.52 |
| Nanfeng | 19.01°N | 109.86°E | 20 | 253.40 | 17347.51 | 2.41 | 23.93 | 137.58 |
| Lianshan | 18.78°N | 110.42°E | 20 | 7.08 | 17550.85 | 2.63 | 25.22 | 135.06 |
| Tianwei | 19.82°N | 110.97°E | 20 | 7.08 | 17550.85 | 2.63 | 25.22 | 135.06 |
| Houhu | 19.63°N | 110.65°E | 20 | 82.40 | 16959.90 | 2.49 | 24.06 | 146.25 |
| Wene | 19.35°N | 110.17°E | 20 | 105.30 | 17252.11 | 2.48 | 24.25 | 142.11 |
| Kanmao | 19.06°N | 109.68°E | 20 | 314.10 | 17383.00 | 2.36 | 23.38 | 134.62 |
| Yongming | 18.78°N | 109.19°E | 20 | 152.80 | 17685.54 | 2.43 | 24.68 | 111.81 |
| Yinggehai | 18.51°N | 108.70°E | 20 | 4.02 | 17779.99 | 2.53 | 25.53 | 103.24 |

**Table S1.** Geographic, environmental information and sample size of sampling sites. Ns = sample size.
