# Supplementary material for: Genetic constraints on in-situ reflectance spectral variation in bermudagrass populations across Hainan Island
Source: Plant Phenomics. 2026 Jan 14;8(1):100168. doi: 10.1016/j.plaphe.2026.100168 (PMC13109577; doi:10.1016/j.plaphe.2026.100168)
Supplement: Multimedia component 2 [file mmc2.docx]

Table S2 Validation metrics of PLS-DA classification among populations and genetic groups.

|  |  | **Classification Accuracy: Mean (SD)** | **F1 Scores: Mean (SD)** | **Cohen’s Kappa Scores: Mean (SD)** |
| --- | --- | --- | --- | --- |
| **Full-spectrum** |  |  |  |  |
| Natural leaf spectral dataset | Among 14 populations | 0.8676 (0.08) | 0.8624 (0.06) | 0.8575 (0.09) |
| Natural canopy spectral dataset | Among 14 populations | 0.4174 (0.06) | 0.4618(0.05) | 0.3725(0.06) |
| Natural leaf spectral dataset | Among 8 groups | 0.8332 (0.01) | 0.8347 (0.03) | 0.8094 (0.02) |
| **Variable importance projection** | |  |  |  |
| Natural leaf spectral dataset | Among 14 populations | 0.7291 (0.02) | 0.7025 (0.06) | 0.7083 (0.07) |
| Natural canopy spectral dataset | Among 14 populations | 0.1394 (0.03) | 0.1468 (0.04) | 0.0731 (0.08) |
| Natural leaf spectral dataset | Among 8 groups | 0.5876 (0.04) | 0.5961 (0.02) | 0.4923 (0.05) |
