# Supplementary figures and images for: Genetic constraints on in-situ reflectance spectral variation in bermudagrass populations across Hainan Island
Source: Plant Phenomics. 2026 Jan 14;8(1):100168. doi: 10.1016/j.plaphe.2026.100168 (PMC13109577; doi:10.1016/j.plaphe.2026.100168)

(a)

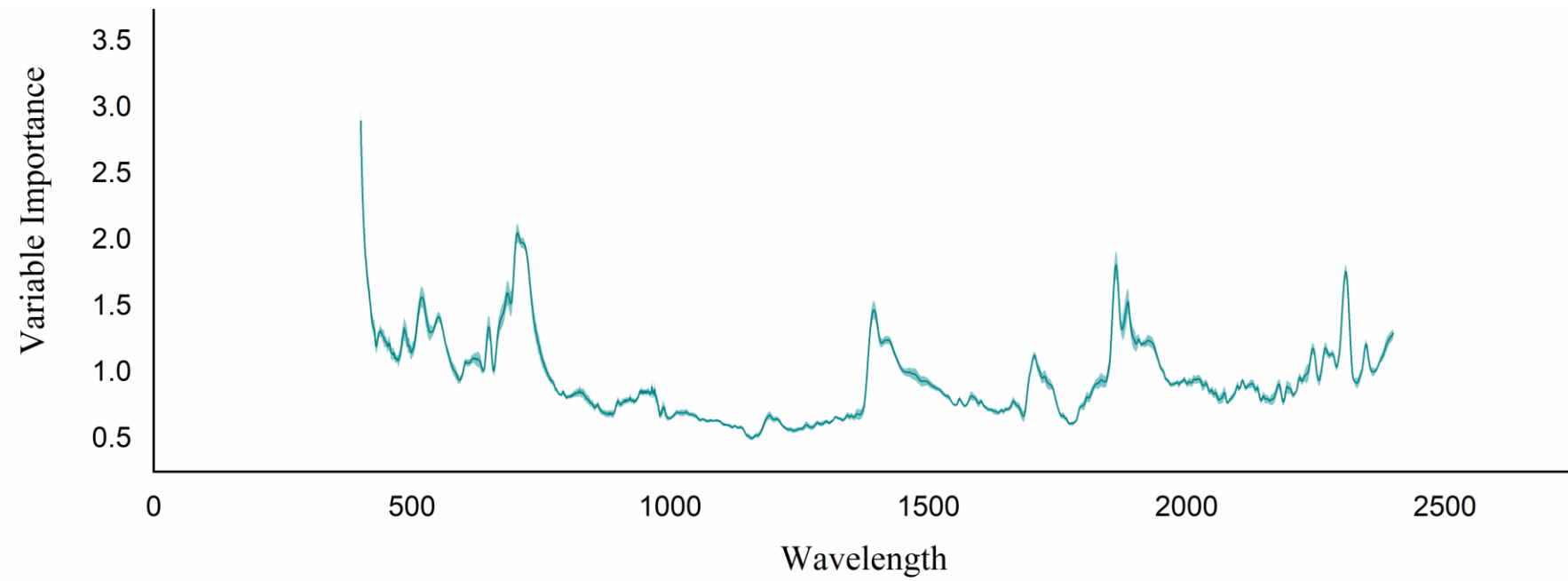

(b)

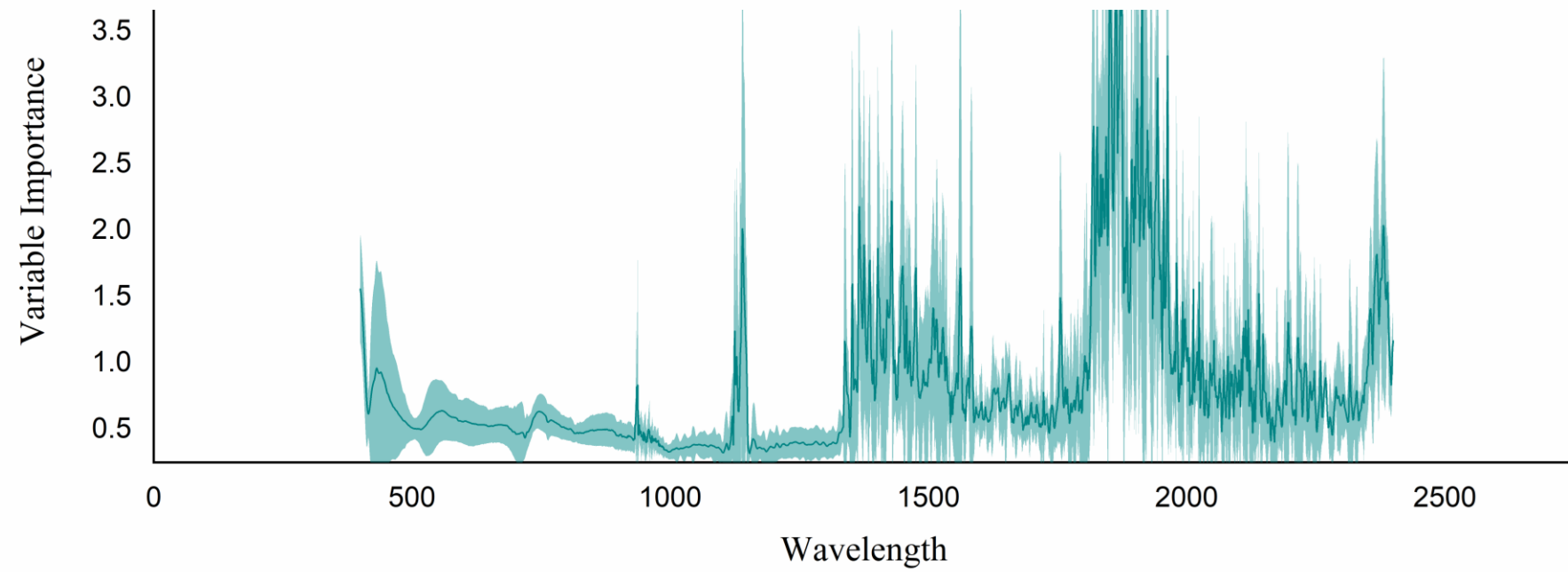

Supplement: Multimedia component 3 [file mmc3.pdf]
